# Preparation and Heavy Metal Adsorption Performance of 2-Aminopyridine-Modified Sodium Alginate/Polyacrylic Acid Hydrogel

**DOI:** 10.3390/gels11040224

**Published:** 2025-03-21

**Authors:** Tingxiang Wu, Amatjan Sawut, Rena Simayi

**Affiliations:** 1 State Key Laboratory of Chemistry and Utilization of Carbon Based Energy Resources, College of Chemistry, Xinjiang University, Urumqi 830017, China; wtxwtx096@163.com; 2 College of Chemical Engineering, Xinjiang University, Urumqi 830017, China

**Keywords:** modified alginic acid, hydrogels, heavy metal adsorption, Schiff base, wastewater treatment

## Abstract

This study utilized the Schiff base reaction as a chemical bonding method to successfully graft 2-aminopyridine onto oxidized sodium alginate, resulting in the formation of modified sodium alginate (OSM). Subsequently, the OSM/polyacrylic acid (OSM/PAA) hydrogel was synthesized via a thermally initiated free radical polymerization process and evaluated as an adsorbent for the removal of heavy metal ions from wastewater. Comprehensive characterization of the prepared samples was performed using FT-IR, SEM, and TGA. The influence of temperature, pH, adsorbent dosage, contact time, and heavy metal ion concentration on the adsorption capacity of the OSM/PAA adsorbent in simulated wastewater was thoroughly investigated. Additionally, a detailed analysis of the adsorption thermodynamics, kinetics, and mechanisms was conducted. Experimental results indicated that at 25 °C, pH 5.0, and an adsorbent dosage of 0.4 g/L, the maximum adsorption capacities of the OSM/PAA hydrogel for Cu(II), Zn(II), and Ni(II) were 367.64 mg/g, 398.4 mg/g, and 409.83 mg/g, respectively. These findings suggest that the adsorption of heavy metal ions by OSM/PAA is a spontaneous, heterogeneous chemical process with significant potential for practical applications in wastewater treatment.

## 1. Introduction

With the rapid progress of modern industry, heavy metal contamination has become one of the most critical global environmental issues [1,2]. Heavy metal waste mainly stems from industries such as metal smelting, fertilizer production, battery manufacturing, dyeing, and electroplating [3,4]. These sources discharge heavy metals into the environment through diverse channels, resulting in severe water pollution [5]. Significantly, heavy metal ions like Cu(II), Zn(II), and Ni(II) are frequently present in industrial effluents [6,7,8]. These ions possess high toxicity, are resistant to be metabolized, and have a propensity to accumulate in living organisms, thus, posing serious threats to both ecological systems and human health [9,10,11]. Therefore, there is an urgent necessity to develop efficient, cost-effective, and environmentally sustainable technologies for treating heavy metal-contaminated wastewater.

Currently, the common methods for treating heavy metal wastewater include chemical precipitation [12], ion exchange [13], and membrane separation [14]. Although chemical precipitation is relatively easy to operate, it produces a large amount of sludge and may lead to secondary pollution [15]. Ion exchange is costly, and the resin regeneration process is complicated [16]. Membrane separation demands advanced equipment and incurs relatively high operating costs [17]. Adsorption-based methods are simple and efficient, and are widely utilized for removing heavy metals [18,19]; however, conventional adsorbents can cause secondary environmental pollution [20]. Hydrogels, a type of high-molecular mass material with a three-dimensional network structure, show remarkable potential in wastewater treatment due to their unique physical and chemical properties, such as high water absorption capacity, excellent biocompatibility, and adjustable pore structures [21,22,23].

The advantages of using hydrogels in the treatment of heavy metal wastewater are obvious. Hydrogels can effectively remove various heavy metal ions through adsorption and are reusable, thereby reducing treatment costs and serving as efficient adsorbents [24,25]. Hydrogels synthesized with biopolymers exhibit excellent biocompatibility, biodegradability, non-toxicity, and safety, leading to their increasing application in heavy metal ion adsorption [26,27,28]. Looking forward, functionalized and composite hydrogels are anticipated to become a research focus by incorporating more active groups or combining with other materials to enhance adsorption capacity [29,30,31]. However, current challenges still exist regarding adsorption capacity, selectivity, and mechanical strength [32]. Many researchers [33,34,35,36] have enhanced the adsorption capacity and selectivity of hydrogels through structural modifications or the introduction of functional groups, while also attempting to improve mechanical properties by incorporating reinforcing materials.

The natural origin and biodegradability of biomass hydrogels make them highly promising for environmental remediation [37]. Their effective water absorption allows the adsorption of various pollutants in aquatic environments, and their abundant internal pores increase the specific surface area available for adsorption [38]. Moreover, these hydrogels can be chemically modified to provide a rich source of active binding sites for heavy metal ions [39]. Sodium alginate, a naturally occurring polysaccharide with abundant sources and excellent biodegradability and biocompatibility, contains a high density of hydroxyl and carboxyl groups, making it a popular choice for hydrogel preparation [40,41]. These characteristics have attracted the attention of researchers who have used them as a starting platform for the development of new materials including adsorbents [42]. However, the adsorption capacity of sodium alginate is limited because its intrinsic functional groups provide relatively few binding sites for heavy metal ions, resulting in a low adsorption capacity for effective removal of high concentrations of heavy metal ions from wastewater [43]. In addition, its poor adsorption selectivity and the lack of specific adsorption capacity of sodium alginate for one or several specific heavy metal ions complicate the precise separation and removal of the target heavy metals in complex wastewater systems containing multiple co-existing ions, thereby reducing its effectiveness in treating specific heavy metal pollution [44]. Thus, sodium alginate containing a large number of hydroxyl groups can be further modified to facilitate optimization of the aforementioned deficiencies.

In this study, to enhance the adsorption capacity and selectivity of alginate-based hydrogels for heavy metals, 2-aminopyridine was used to modify alginate. The 2-aminopyridine molecule contains nitrogen and other active groups, which can bind to oxidized alginate through the Schiff base, thus, producing modified alginate (OSM). The modified alginate has rich N, O atoms, which can effectively adsorb heavy metal ions in aqueous solutions. Furthermore, OSM was compounded with polyacrylic acid (PAA) by a thermal-initiated radical polymerization to form OSM/PAA hydrogels. The structure and performance of the hydrogels were deeply analyzed by various characterization techniques such as FT-IR, SEM, and TGA, and the effects of temperature, pH value, adsorbent dosage, adsorption time, and heavy metal ion concentration on the adsorption of heavy metal ions by the hydrogels were systematically studied. The adsorption thermodynamics, kinetics, and mechanism were also explored. This research aims to provide a novel and efficient adsorbent material for heavy metal wastewater treatment and offer valuable insights for related studies.

## 2. Results and Discussion

### 2.1. FT-IR and XPS Analysis

**Figure 1:**
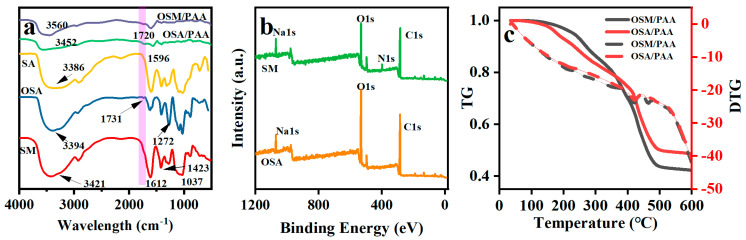
(**a**). FTIR spectra of SA, OSA, SM, OSA/PAA, OSM/PAA; (**b**). XPS spectra of OSA, SM; (**c**). TGA, DTG analysis of OSA/PAA and OSM/PAA.

The various functional groups in the modified sodium alginate and hydrogel were measured by FTIR as shown in Figure 1a. The oxidation of SA to OSA was verified by FT-IR spectroscopy of sodium alginate and oxidized sodium alginate. Due to the absorption peak of OSA, a new absorption peak corresponding to the characteristic band of the carbonyl group of OSA appeared at 1731 cm^−1^, showing the successful preparation of OSA [45,46]. The degree of oxidation of sodium alginate is about 35%, as determined by titration with hydroxylamine hydrochloride. The weak peak at 1731 cm^−1^ is likely due to the formation of hemiacetal between aldehyde and hydroxyl groups, which reduces the intensity of the carbonyl peak [47,48]. The formation of modified SM occurs through a Schiff base reaction between the amino group of 2-aminopyridine and the aldehyde group of OSA. This was obtained in the FTIR spectrum, which exhibited an absorption band at 3394 cm^−1^ attributed to the hydroxyl (-OH) stretching vibration of OSA [49]. In SM, the peak absorption is redshifted from 3394 cm^−1^ to 3421 cm^−1^ and the peak area is broadened, which is related to the formation of -C=N- [5,46,50]. A sharp peak at 1612 cm^−1^ and 1423 cm^−1^ are attributed to the -C-N stretching vibration and the single-bond bending vibration corresponding to the C-N [51,52]. As shown in Figure 1a for OSM/PAA, the characteristic peak of acrylic acid is still present in the hydrogel. In addition, the peak at 1596 cm^−1^ is attributed to the stretching of the C=N double bond [53] and the strong peak at 1720 cm^−1^ is attributed to the stretching of the C=O double bonding moiety in the OSA/PAA [49], where the C=O double bond is weakened and shifted after the reaction. In addition, the peak corresponding to the O-H single bond group shifted to 3452 cm^−1^ at 3560 cm^−1^, overlapping with the N-H single bond. The above results show that PAA and OSM successfully form a dual network structure by means of free radical polymerization.

### 2.2. TGA

The thermogravimetry of the samples is shown in Figure 1c between room temperature and 147 °C for the loss of water in the hydrogel. Between 147 °C and 288 °C, the weight loss of bound water in the hydrogel was about 12.3%. A temperature range of 288–423 °C is the decomposition of the OSM network with a weight loss of about 20.03%. A temperature range of 423–496 °C is the decomposition of the cross-linker in polyacrylic acid with an approximate weight loss of 25.11%. A temperature range of 496–600 °C is the carbonization of small molecules such as the carboxyl groups of MA, AA, and 2-aminopyridine, with a final residual organic ash fraction of about 42.26% by weight. They exhibit very close initial decomposition temperatures, and the peak height of the first DTG peak of OSM/PAA is even slightly lower than that of OSA/PAA. These indicate that the introduction of OSA into 2-aminopyridine does not negatively affect the thermal stability.

### 2.3. SEM

**Figure 2:**
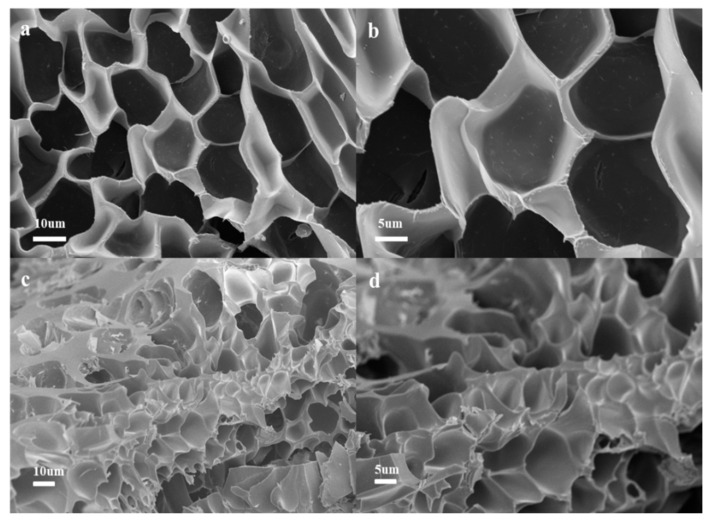
SEM images of OSM/PAA hydrogel before (**a**,**b**) and after (**c**,**d**) adsorption.

After the two hydrogels were immersed in distilled water and Cu(II) ion solution, respectively, and swelled to twice the original volume, they were rapidly cooled to −80 °C with liquid nitrogen and freeze-dried for 72 h. The morphology was observed by SEM to obtain the photographs shown in Figure 2. The cross-sectional SEM photograph of the hydrogel immersed in distilled water is shown in Figure 2a,b, in which it can be clearly seen that the hydrogel has good and dense pores. Such a pore structure can easily allow metal ions to enter the interior of the hydrogel, which in turn greatly increases the adsorption capacity of the hydrogel. Figure 2c,d is a cross-sectional SEM photograph of a hydrogel soaked with Cu(II) solution. Figure 2c shows the obvious heavy metal filling of the pore structure in the hydrogel, and the width of the pore structure becomes smaller because Cu^2+^ enters into the interior of the hydrogel to form a ligand chelate with the modified sodium alginate or electrostatic interaction with the carboxylate inside the hydrogel, leading to a smaller pore size of the hydrogel network. Figure 2d shows that the pores of the hydrogel disappear due to the entry of Cu^2+^ into the hydrogel pores because of the metal ions adsorbed on the pore walls [17,54].

Based on the Figure S4 results, the nitrogen (N) content is 2.22%, which corresponds to a 2-aminopyridine mass percentage of approximately 7.45%. This calculation is derived from the fact that nitrogen constitutes 29.78% of the 2-aminopyridine molecule (C_5_H_6_N_2_).

### 2.4. Synthesis Conditions and Properties of OSM/PAA Hydrogels

#### 2.4.1. Selection of the Optimal Synthesis Conditions

Subtle changes in the polymerization conditions can lead to significant alterations in the structure and adsorption capacity of the synthesized hydrogels. In this study, we investigated the effects of cysteine content (Cys, which is cysteamine dihydrochloride and mainly functions to oxidize sodium alginate for cross-linking to form the first cross-linked network), acrylic acid (AA) concentration, neutralization degree (ND) of acrylic acid, ammonium persulfate (APS, initiator), and N,N′-dimethylacrylamide (MBA, cross-linking agent) on the Cu^2+^ adsorption capacity of OSM/PAA hydrogels.

As depicted in Figure S1a, when the Cys content increases from 3% to 6%, the adsorption capacity of the hydrogel for Cu^2+^ rises significantly. However, once the Cys content exceeds 6%, the adsorption capacity starts to decline. Cys mainly functions in the cross-linking of oxidized sodium alginate to form the first cross-linked network. An appropriate amount of Cys promotes the formation of a favorable cross-linked network for adsorption. Nevertheless, an excessive amount may disrupt the network structure or cause other side reactions, thereby reducing the adsorption ability. This indicates that there is an optimal range for Cys content in the synthesis of OSM/PAA hydrogels to achieve high-efficiency Cu^2+^ adsorption. From Figure S1b, it is evident that as the concentration of AA (acrylic acid) increases from 20% to 30%, the adsorption capacity gradually increases. But when the concentration continues to rise beyond 30%, the adsorption capacity decreases. An increase in AA concentration can potentially increase the number of functional groups in the hydrogel that can interact with Cu^2+^. However, an overly high concentration may lead to excessive cross-linking of polymer chains or an unfavorable spatial structure that hinders the access of Cu^2+^ to the adsorption sites. This implies that precise control of AA concentration is crucial for optimizing the adsorption capacity of the hydrogel. Figure S1c shows that as the neutralization degree of acrylic acid increases from 65% to 75%, the adsorption capacity shows a downward trend, and it remains relatively stable between 75% and 80%. The change in the neutralization degree affects the charge properties and spatial structure of the hydrogel. A too-high neutralization degree may not be conducive to the adsorption of Cu^2+^, possibly due to changes in the electrostatic interaction or the swelling behavior of the hydrogel, which in turn affects the accessibility of Cu^2+^ to the adsorption sites. According to Figure S1d, as the APS content increases from 0.005 to 0.01, the adsorption capacity rises rapidly. However, when it exceeds 0.01, the adsorption capacity drops sharply. APS initiates the polymerization reaction to form the hydrogel network. An appropriate amount of APS can lead to the formation of a suitable network structure for Cu^2+^ adsorption. But an excessive amount makes the polymerization reaction too intense, resulting in an unreasonable network structure and a decline in adsorption capacity. This emphasizes the importance of accurately controlling the amount of initiator in the synthesis process. As shown in Figure S1e, as the MBA content increases from 0.01 to 0.03, the adsorption capacity continues to increase. After 0.03, the adsorption capacity tends to level off. An appropriate increase in MBA content can optimize the cross-linking structure of the hydrogel, enhancing its adsorption ability. Once the cross-linking reaches a certain level, further increasing the MBA content has little impact on the adsorption capacity. This suggests that there is a saturation point for the cross-linking effect of MBA on the adsorption capacity of the hydrogel.

Based on the comprehensive analysis of all figures, when the Cys content is 6%, the AA concentration is 30%, the neutralization degree of acrylic acid is 65%, the APS content is 0.01, the MBA content is 0.03, and the adsorption capacity of the hydrogel for Cu^2+^ is relatively high within the respective factor investigation ranges. These conditions can be tentatively considered as the optimal synthesis conditions to endow the OSM/PAA hydrogel with good Cu^2+^ adsorption capacity. However, further multi-factor interaction experiments and in-depth research are still needed to accurately determine the absolute optimal synthesis conditions, as the actual situation may be affected by complex interactions among multiple factors.

#### 2.4.2. Swelling Behavior of OSM/PAA Hydrogels

**Figure 3:**
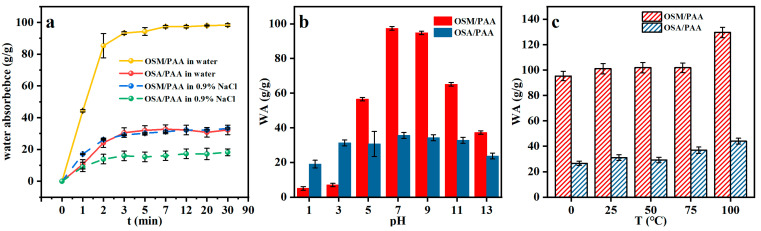
(**a**) Effect of time on hydrogel swelling, (**b**) effect of pH on hydrogel swelling, and (**c**) effect of temperature on hydrogel swelling.

The use of hydrogel in industrial, agricultural, and medical applications is an important indicator of its swelling properties. Liquid–solid contact time is one of the parameters of hydrogel in absorptive swelling. Figure 3a shows that OSM/PAA hydrogel adsorbs water more rapidly than OSA/PAA hydrogel and the hydrogels in 0.9% NaCl; this may be due to the larger osmotic pressure gradient of the polyelectrolyte OSM-g-AA-Na. It was also observed that both the rate of water uptake of this hydrogel and the rate of water uptake in 0.9% NaCl solution increased rapidly within 2 min, saturated slowly after 4 min, and reached equilibrium within 10 min, suggesting that the rate of dissolution was unaffected, despite the decrease in equilibrium uptake due to the osmotic environment of 0.9% NaCl. The hydrogel synthesized in this study constitutes a weak electrolyte system containing ionizable groups. Owing to partial ionization, the degree of ionization varies with pH fluctuations, as illustrated in Figure 3b, consequently inducing corresponding swelling or shrinkage behavior in the hydrogel. From Figure 3c, it can be seen that the swelling of the hydrogel does not change much with temperature, indicating that the hydrogel has good thermal stability, and at the same time, this is consistent with the results obtained from the previous TG.

Through Figure 3a–c, it is found that the swelling property of OSM/PAA is one third of that of OSA/PAA, which may be due to the fact that the modified sodium alginate has more free functional groups and a smaller cross-linking degree, which makes OSM/PAA have better swelling behavior. In Figure 3b, it can be seen that the water absorption of OSA/PAA is more stable in response to pH changes, presumably because the hydrogel does not contain 2-aminopyridine, which is pH-sensitive, resulting in a lesser effect of pH on the hydrogel pairs.

#### 2.4.3. Comparison of OSA/PAA and OSM/PAA Adsorption Capacity

**Figure 4:**
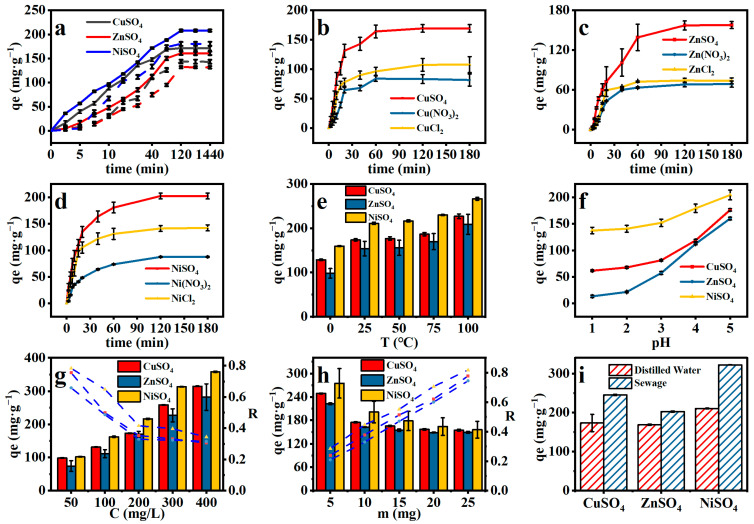
(**a**) Adsorption of Cu(II), Zn(II), Ni(II) by different hydrogels, solid line is OSM/PAA and dashed line is OSA/PAA; (**b**) Comparison of adsorption of Cu(II) by OSM/PAA under different anions; (**c**) Comparison of adsorption of Zn(II) by OSM/PAA under different anions; (**d**) Comparison of adsorption of Ni(II)adsorption comparison. (**e**) Comparison of adsorption of Cu(II), Zn(II), and Ni(II) by OSMPAA at different pH adsorption; (**f**) Different temperatures; (**g**) Relationship between adsorption capacity and initial metal ion concentration; (**h**) Different amounts of adsorbents; (**i**) Adsorption experiments with real sewage and distilled water as solvents. Unless otherwise specified, for the adsorbent: m = 15 mg, for the metal ion solution: V = 25 mL, c = 200 mg·L^−1^, t = 12 h.

In this experiment, thermal initiation was used and the collision of free radicals between monomers occurred at a certain temperature. The results are shown in Figure 4a by comparing the adsorption of two kinds of hydrogels before and after modification. The adsorption capacity of the modified hydrogel was more superior, presumably due to the introduction of Schiff base bonds, which can chelate with metal ions and, therefore, have a greater effect on the adsorption of the hydrogel.

#### 2.4.4. Effect of Anions on Adsorption

The effect of different anions on the adsorbent can be seen in Figure 4b–d, where sulfate has a much higher degree of adsorption of heavy metal ions compared to chloride and nitrate. The adsorption of heavy metals by the adsorbent in nitrate and chloride solutions was similar, probably due to the fact that sulfate has a different valence state than chloride and nitrate. The adsorption trends of Cu(II), Zn(II), and Ni(II) remain consistent in Figure 4 [55]. Indeed, sulfate ions may promote metal adsorption due to the generally lower solubility of metal sulfates compared to chlorides or nitrates. Additionally, sulfate ions can participate in co-adsorption to maintain charge balance [56]. It increased sharply within 10 min, and the contact time reached adsorption equilibrium within 60 min, which is presumed to be due to the fact that the adsorption capacity of the OSM/PAA adsorbent was not saturated in the initial stage due to the insufficient adsorption time, resulting in a rapid increase in the amount of adsorption with time. This is consistent with the performance of water absorption of OSM/PAA. When the adsorption time was prolonged, the main adsorption sites of OSM/PAA adsorbent were gradually occupied by heavy metal ions until saturation, and the adsorption capacity was slowly increased to reach equilibrium [57]. The adsorption time of this is adsorbent is shorter compared to other reported adsorbents [51].

#### 2.4.5. Effect of pH and Temperature on Adsorption

The effect of pH on the adsorption capacity of Cu(II), Zn(II), and Ni(II) was investigated in Figure 4e. When the initial pH is >5, metal hydroxides precipitate out of solution, which may affect the accuracy of the results [52]. In Figure 3a, the adsorption capacity increases with increasing solution pH, which can be attributed to the competition between metal ions and high concentrations of hydrogen ions at low pH levels when metal ions bind to the adsorbent [58]. Also, the main adsorption sites are protonated at low pH and the surface of the adsorbent is positively charged, which reduces the stability and number of electrostatic interactions between the hydrogel and heavy metal ions. At the same time, the low pH reduces the swelling capacity of the adsorbent, resulting in fewer heavy metal ions entering the interior of the hydrogel [51]. All things considered, pH = 5 is the appropriate choice. The maximum K_d_ values of the three heavy metal ions are K_d_^Ni^ = 1.25 × 10^8^ mL/g, K_d_^Cu^ = 2.80 × 10^7^ mL/g, and K_d_^Zn^ = 9.68 × 10^6^ mL/g, and when the K^d^ is greater than 1.0 × 10^4^, it can be considered to be available as an adsorbent [59,60]. Combined with the adsorption rate and K. value, it can be seen that OSM/PAA has the best adsorption effect on Ni^2+^, followed by Cu^2+^, and Zn^2+^ is the worst.

#### 2.4.6. Effect of Metal Concentration and Dosage on Adsorption

Figure 4g shows the effect of initial metal ion concentration on the adsorption of heavy metal ions by the hydrogel. The adsorption capacity of the adsorbent increased significantly when the initial concentration increased. This phenomenon is explained by the fact that the amount of OSM/PAA adsorbent and the number of adsorption sites remain constant. The initial adsorption concentration is positively correlated with the adsorption capacity; therefore, an increase in metal concentration leads to a corresponding increase in adsorption capacity. However, the adsorption capacity is negatively correlated with the removal rate, and when the solution concentration is continuously increased, the excess contaminants will not be actively adsorbed by the OSM/PAA adsorbent, and the adsorption equilibrium is reached mainly through the osmotic pressure and concentration difference [57]. In summary, a metal solution with a concentration of 200 mg/g was chosen as the initial concentration for this experiment.

From Figure 4h, it can be seen that the adsorption capacity decreased significantly with the increase in adsorbent dosage, and the adsorption capacity of OSM/PAA for Cu(II), Zn(II), and Ni(II) decreased from 248 mg/g to 155 mg/g, 221 mg/g to 148 mg/g, and 285 mg/g to 161 mg/g, respectively, with the increase in the adsorbent dosage from 0.2 g/L to 1 g/L to 161 mg/g, 221 mg/g to 148 mg/g, and 285 mg/g to 161 mg/g, respectively. Due to the increase in adsorption active sites as a result of increasing the adsorbent dosage, the M^2+^ adsorbed per unit of mass of OSM/PAA decreases when the total amount of metal ions in solution remains unchanged, and the degree of unsaturation of the adsorbent’s active sites increases, resulting in a significant decrease in the adsorption capacity. In addition, the increase in the adsorbent dosage also leads to the accumulation of adsorbent particles and the increase in the adsorption diffusion pathway, thus, decreasing the adsorption capacity. Therefore, the adsorbent dosage of 0.4 g/L was selected to determine the maximum adsorption capacity of OSM/PAA.

#### 2.4.7. Realistic Wastewater Simulation Experiments

In a comparison of a tapwater and distilled water matrix, tap water was used as the solvent. Figure 4i shows the adsorption of Cu(II), Zn(II), and Ni(II) by the composite hydrogel in different solvents. The experimental results showed that the metal adsorption in tap water was higher than that in distilled water. This is due to the fact that there are more free ions in tap water, which will increase the adsorption capacity of the hydrogel. As in high concentrations, the adsorbent itself has a capacity to adsorb, but the adsorbent “stops working” when the ion concentration (or ionization of the solution) falls below a certain level. It is also possible that the pH of the tap water is more suitable for adsorption. Tap water is usually treated to a pH close to neutral and alkaline, whereas distilled water has an acidic pH, which is consistent with the previous conclusion in Figure 4f.

#### 2.4.8. Competitive Adsorption

Multiple heavy metal ions are usually present in water. Therefore, this study investigated the adsorption properties of hydrogels in complex heavy metal ion solutions. Adsorption experiments were carried out by placing the hydrogel in a two-by-two mixture of (Cu(II), Zn(II), and Ni(II)) at 400 mg/L 12.5 mL. As shown in Figure S4, the adsorption capacity of the hydrogel for Cu(II) was significantly higher than that for Zn(II) in the binary Cu(II)/Zn(II) system, which is a common trend in adsorption studies. This phenomenon can be attributed to the relatively small ionic radii of Cu^2+^ (0.073 nm) and Zn^2+^ (0.074 nm), which, combined with their high charge densities, enable these ions to exert stronger electrostatic interactions with the lone pair electrons in the adsorbent, thereby facilitating the formation of more stable complexes [61]. In the Cu(II)/Ni(II) system, the adsorption capacity of Cu(II) on Cu(II) remains stable, while the adsorption capacity of Ni(II) is relatively high. It is noteworthy that the adsorption capacity of Zn(II) in the Zn(II)/Ni(II) system was significantly lower than that of the single ion system. This phenomenon suggests that Zn(II) may have a competitive adsorption mechanism with other metal ions, leading to a significant decrease in its adsorption capacity in a multi-ion coexistence environment [62,63].

#### 2.4.9. Effect of Temperature on Adsorption

From Figure 4f, it can be seen that although the adsorption capacity of the OSM/PAA hydrogel is not very significant to temperature, it changes slightly only when the temperature change is relatively large, which may be due to the fact that this adsorbent is thermally initiated, so the temperature does not have a significant effect on this adsorbent. The metal ion adsorption experiments were observed on the prepared adsorbents at three temperatures, 298, 323, and 348 K. The adsorption process was carried out by the adsorbent. Since the adsorption process is a heat absorbing process, an increase in temperature can shift the adsorption equilibrium towards strong adsorption [50].

In order to study the thermodynamics, the data obtained were fitted by Eq. The results are shown in Figure S2 and the corresponding thermodynamic parameters ΔG^o^, ΔS^o^, and ΔH^o^ are shown in Table 1.

Based on the data in the table, the slopes and intercepts of the curves of lnKc versus 1/T allow us to calculate the thermodynamic parameters (ΔH°, kJ/mol) and (ΔS°, J/K mol), respectively. The positive values of enthalpy confirm that the adsorption of all three metal ions by the prepared adsorbents is a heat-absorbing process. This result indicates that the qe value can be increased by temperature. In addition, the entropy values are also positive. Positive values indicate that the adsorbent and adsorbate interfaces are more disordered during the adsorption process. Finally, the negative value of free energy Gibbs (ΔG°, kJ/mol) indicates that the conclusion is correct and that the adsorption of metal ions on the adsorbent is spontaneous.

### 2.5. Adsorption Kinetics

Contact time between adsorbent and heavy metals is an important factor in understanding the adsorption mechanism [64]. From Figure 4, it can be seen that the adsorption capacity of the hydrogel increased rapidly at the beginning of the adsorption and rose slowly until equilibrium in the following time, and the adsorption capacity of the adsorbent reached saturation at 30 min of adsorption. To further investigate the adsorption mechanism, the relationship between adsorption capacity and metal ion concentration was discussed using adsorption kinetics. In this study, quasi-primary and quasi-secondary kinetic models ((3), (4)), which are commonly used to characterize the adsorption kinetics of metal ions, were used to investigate the adsorption mechanism.

**Figure 5:**
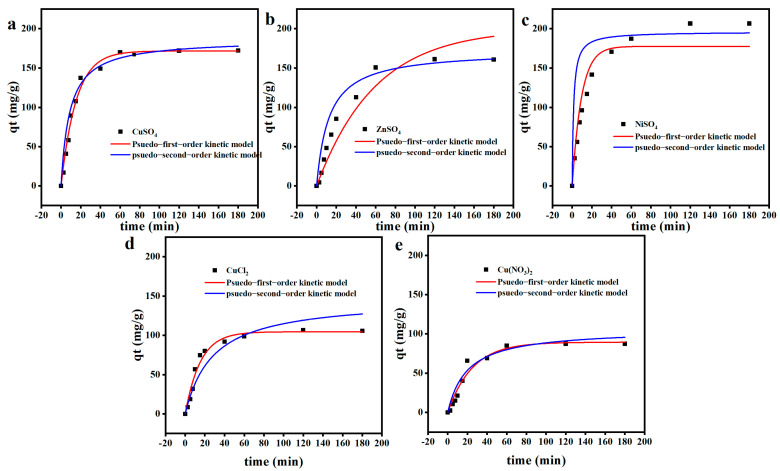
Pseudo first-order dynamics and pseudo-secondary dynamics curves of OSM/PAA hydrogels (**a**) CuSO_4_, (**b**) ZnSO_4_, (**c**) NiSO_4_, (**d**) CuCl_2_, and (**e**) Cu(NO_3_)_2_.

Model comparison was performed by computing the values of the coefficient of determination R2 and the average absolute relative deviation (AARD) which provided additional information about the quality of fitting [49]. The results for these linear fits are summarized in Figure 5 and Table 2. The correlation parameters R^2^ of the pseudo-primary kinetic model for Cu(II), Zn(II), and Ni(II) ions were 0.9877, 0.9113, and 0.9269, respectively, which were lower than the corresponding pseudo-secondary kinetic parameters R^2^ (0.9981, 0.9940, and 0.9852). This indicates that the quasi-secondary kinetic model is more suitable for describing the adsorption behavior of the hydrogel when absorbing heavy metal ions in solution, which suggests that the adsorption process is mainly chemisorption. However, further characterization is required to confirm the nature of the adsorption process [65].

### 2.6. Adsorption Isotherms

In order to further investigate the correlation between initial elemental concentration of pollutants and adsorption, and to probe the interaction behavior between adsorbents and pollutants, the adsorption isotherms of metal ions were detected by Langmuir, Freundlich, Temkin, and Dubinin–Radushkevich isotherms (Figure S3) [57].

Parameters obtained by Langmuir, Freundlich, Temkin, and Dubinin–Radushkevich isotherm models are shown in Table 3. By comparing the data in the table, it is clear that the adsorption of heavy metal ions by OSM/PAA is more in agreement with the Freundlich linear fit. The correlation coefficient R2 is better than the correlation coefficients of other isothermal models. This suggests that the modeling of adsorption of heavy metal ions by OSM/PAA is mainly based on the Freundlich theory of adsorption and we can postulate that the mechanism is chemisorption. In addition, the maximum adsorption molecular weights of OSM/PAA for Cu(II) Zn(II) Ni(II) can be known from the Langmuir model to be 367.64, 398.4, and 409.83 mg/g, respectively. Based on the parameter 1/n < 1 in the Freundlich, it can be inferred that the adsorption process is a spontaneous reaction in a non-homogeneous phase. The adsorption data fitted well with the Freundlich model, which is widely used to describe adsorption processes with multilayer characteristics [66]. However, as an empirical model, it does not provide definitive conclusions about the adsorption mechanism. Based on the fitting results of the thermodynamic and kinetic models, the characteristics of the whole adsorption process can be summarized as follows: (a) heat adsorption is confirmed by the enthalpy change in the thermodynamic parameter (ΔH° > 0); (b) the non-homogeneous phase adsorption mechanism is supported by the Freundlich isotherm model (n ≠ 1) and by the high goodness of fit of the quasi second-order kinetic model (R^2^ > 0.99); and (c) the spontaneity is indicated by the negative value of Gibbs free energy change (ΔG° < 0) as indicated by the negative values.

### 2.7. Repeatability Testing of Hydrogels

**Figure 6:**
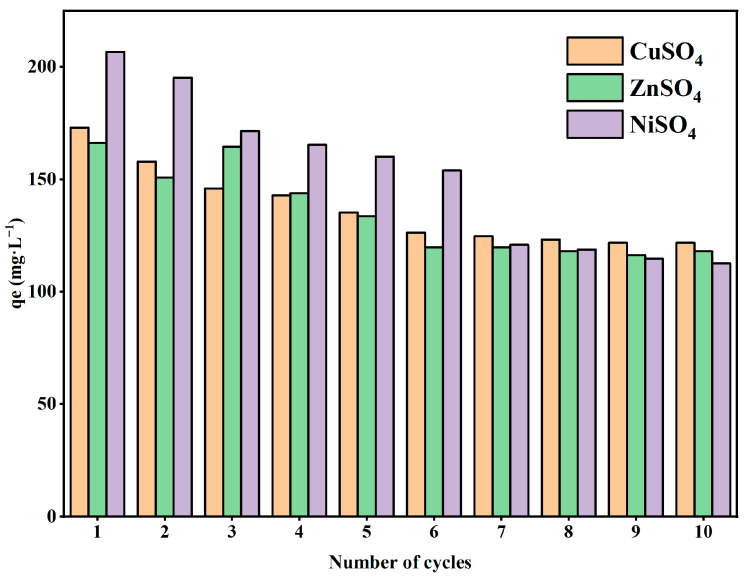
OSM/PAA hydrogel after adsorption–desorption 10 times.

In general, good reproducibility of the binding characteristics is an important indicator to fulfill the low-cost requirement of the adsorbent. The desorption–adsorption process in different ionic solutions with different cycle times is shown in Figure 6. After 10 cycles, the adsorption capacity of the adsorbent for Cu^2+^, Zn^2+^, and Ni^2+^ decreased from 173 mg/g, 166 mg/g, and 206 mg/g to 120 mg/g, 117 mg/g, and 112 mg/g, respectively. With the increase in the number of cycles, the adsorption capacity decreases. This may be due to the influence of desorbing agent HCl during the recirculation of the OSM/PAA, in which the structure was damaged. Secondly, residual desorbing agents (e.g., acidic solutions) or incompletely desorbed metal ions may remain in the hydrogel, potentially occupying active adsorption sites and thereby reducing its adsorption capacity. However, it still has half of the original adsorption capacity, indicating that OSM/PAA has good reusability and regeneration reuse potential.

The adsorption capacity of the prepared OSM/PAA adsorbent was higher than that of most of the reported adsorbents under the same conditions as compared to other reported adsorbents with similar backbones (see Table 4).

### 2.8. Adsorption Mechanism of OSM/PAA Hydrogels

**Figure 7:**
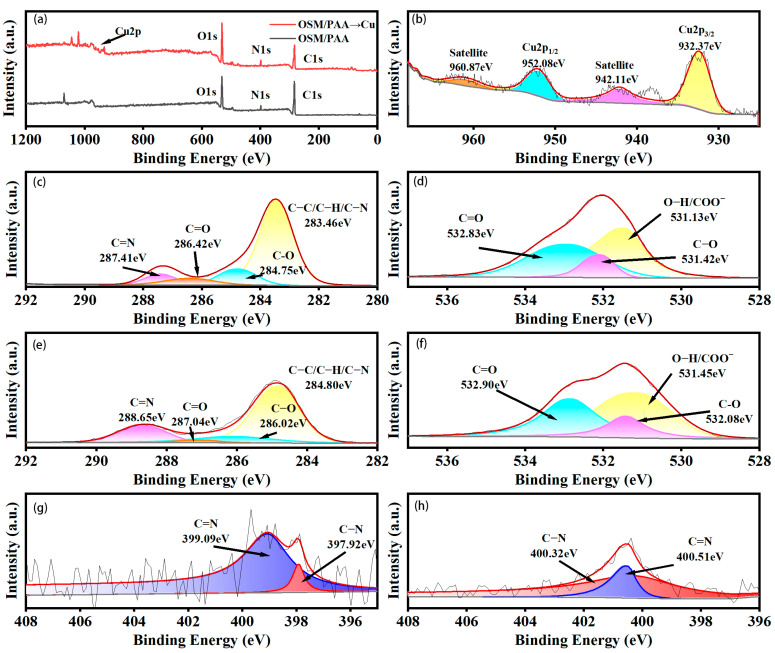
(**a**) XPS survey of OSM/PAA before and after adsorption, (**b**) XPS spectra of OSM/PAA after adsorption of high-resolution Cu2p, and (**c**–**h**) XPS spectra of c1s, o1s, and n1s before and after adsorption.

In order to explore the adsorption mechanism, the interaction between functional groups and metal ions was analyzed using XPS technique. A new peak corresponding to Cu2p at 932 eV can be seen in the OSM/PAA and OSM/PAA-Cu(II) spectra by comparison. In the high-resolution Cu2p spectra, the two peaks at 932.37 eV and 952.08 eV belong to Cu2p_3/2_ and Cu2p_1/2_, respectively, and the two neighboring satellite bands are 942.11 and 960.87 eV, respectively (Figure 7c). The results provide further evidence of the binding of Cu(II) to OSM/PAA [66].

**Figure 8:**
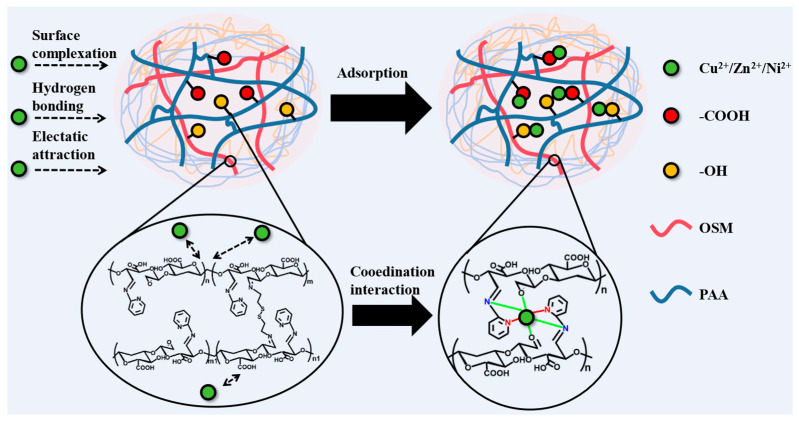
OSM/PAA adsorption mechanism diagram.

In Figure 7c,e, the C1s peaks at 287.41, 286.42, and 284.75 eV were clearly attributed to C=N, C=O, and C-O bonds in OSM/PAA [72], and the binding energies were increased to 288.65, 287.04, 286.02, and 284.8 eV, respectively. In Figure 7d,f, on the O1s spectrum, the peaks at 531.13, 531.42, and 532.83 eV belong to the O-H, C-O, and C=O bonds, respectively. In Figure 7h, after Cu(II) adsorption, the characteristic peaks were shifted to 531.45, 532.08, and 532.90. The change in binding energies proved that carboxyl groups and hydroxyl groups are involved in the complexation of Cu(II). The two peaks in the N1s spectrum at 397.92 and 399.09 eV belong to C-N and C=N bonds, respectively, and the peaks are shifted to 400.51 and 400.32 eV after adsorption of Cu(II). The results indicate that the amine group on the graft can chelate with Cu(II). In summary, the amino, hydroxyl, and carboxyl groups in SM/PAA are involved in the adsorption of heavy metals, and the adsorption mechanism is coordination. During ion exchange and electrostatic interactions, on the one hand, Cu(II) can be adsorbed in the hydrogel by electrostatic adsorption; on the other hand, it can bind to the 2-aminopyridine of SM through a coordination reaction. We hypothesize the ligand complexation mechanism in Figure 8.

## 3. Conclusions

This study developed an eco-friendly modified sodium alginate (OSM) by partially oxidizing sodium alginate with sodium periodate and condensing it with 2-aminopyridine. A hydrogel was prepared via one-pot polymerization with acrylic acid and a cross-linking agent, and its adsorption of heavy metals (Cu(II), Zn(II), Ni(II)) was evaluated. The polymers were characterized using FT-IR, XPS, SEM, and TGA. Batch experiments examined factors like pH, contact time, ion concentration, adsorbent amount, and temperature. OSM/PAA showed maximum adsorption capacities of 367.64, 398.4, and 409.83 mg/g for Cu(II), Zn(II), and Ni(II), respectively. The adsorption followed the Freundlich model and quasi-secondary kinetics, driven by electrostatic interactions and N coordination. Also, anions have an effect on the adsorption capacity, especially in sulfate solutions, which can be adsorbed more. Effective metal desorption and adsorbent recovery from acidic solutions saw more than half of the original adsorption capacity remaining in 10 cycles. OSM/PAA demonstrates high adsorption capacity and reusability but requires further research on efficiency, selectivity, and scalability for industrial applications.

## 4. Experimental Section

### 4.1. Materials

Sodium alginate ((SA, Mw = 100–200,000 Da, purity > 99%) Tianjin Bailun Biotechnology Co., Ltd., Tianjin, China), Sodium periodate (SP, Tianjin Yongrui Fine Chemical Co., Ltd., Tianjin, China), Cystamine dihydrochloride (Cys), 2-Amino pyridine (M), Acrylic acid (AA), (Shanghai McLean Biochemical Science & Technology Co., Ltd., Shanghai, China), Sodium hydroxide (Tianjin Zhiyuan Chemical Reagent Co., Ltd., Tianjin, China), Ethanol (Tianjin Zhiyuan Chemical Reagent Co., Ltd., Tianjin, China), ethanol (Tianjin Zhiyuan Chemical Reagent Co., Ltd., Tianjin, China), N,N′-methyl allyl ether (Tianjin Zhiyuan Chemical Reagent Co., Ltd., Tianjin, China), ammonium persulfate (APS, Tianjin Zhiyuan Chemical Reagent Co., Ltd., Tianjin, China), copper sulfate pentahydrate, zinc sulfate heptahydrate, copper(II) chloride dihydrate, nickel(II) chloride hexahydrate, copper(III) nitrate trihydrate (Tianjin Xinbute Chemical Co., Ltd., Tianjin, China), Nickel Sulfate Hexahydrate, Nickel Nitrate Hexahydrate, Zinc Nitrate Hexahydrate (Shanghai Aladdin Biochemical Science and Technology Co., Ltd., Shanghai, China) Diethyl Dithiocarbamate Sodium Salt Trihydrate (DDTC, Rhône Reagent), Dimethylphenol Orange (XO, Shanghai Shanpu Chemical Co., Ltd., Shanghai, China). All chemicals were sourced from commercial suppliers in China.

### 4.2. Oxidation of Sodium Alginate

OSA was synthesized in accordance with the previously described method [49,72]. Precisely, 1.0 g of SA and 0.5 g of SP were dissolved in 100 mL of phosphate-buffered saline (PBS, pH 5.0) and stirred continuously for 5 h at 25 °C under dark conditions. 1 mL of ethylene glycol was then added to halt the reaction. Subsequently, the reaction mixture was dialyzed against deionized water for 3 days by employing a dialysis bag with a molecular weight cut-off of 3500 Da to eliminate the unreacted compounds. The resultant solution was freeze-dried to obtain OSA, which was subsequently stored at −20 °C for later utilization.

The degree of oxidation (DO) of aldehydes was quantified using the previously reported hydroxylamine hydrochloride method [73]. Specifically, 0.1 g of lyophilized OSA was added to 25 mL of a 0.25 mmol/L hydroxylamine hydrochloride-methyl orange solution, mixed thoroughly to ensure complete dissolution, and reacted under dark conditions for 2 h. Upon completion of the reaction, the amount of HCl generated was determined by titration with a 0.1 mol/L standard NaOH solution. The reaction equation is as follows:

-(CHO)_n_ + nNH_2_-OH-HCl ==== (CH=N-OH)_n_ + nH_2_O + nHCl (1)

The titration was stopped when the color of the solution changed from red to yellow (pH = 5), and the aldehyde group concentration was calculated from the amount of NaOH that had been consumed.

The OD of OSA is calculated as follows in Equation (2):

OD(%) = (V × 0.001 × nNaOH/2)/(m/400) × 100%(2)

### 4.3. Synthesis of Sodium 2-Aminopyridine Alginate (OSM)

After dispersing OSA (0.5 g) and an excess of (0.5 wt%) 2-aminopyridine (M) in 50 mL of distilled water using magnetic stirring at 30 rpm, the pH of the solution was adjusted to 5. The mixture was then stirred in the dark for 8 h. Subsequently, the solution was dialyzed against a pH 5 buffer solution for 2 days, followed by 1 day of dialysis in distilled water. Finally, the solution within the dialysis bag was freeze-dried at −4 °C for 48 h.

### 4.4. Synthesis of OSM/PAA and OSA/PAA Hydrogels

**Figure 9:**
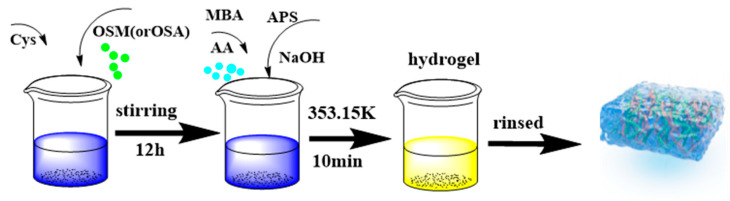
Hydrogel preparation process.

OSM/PAA hydrogels were synthesized according to the following procedure: an appropriate amount of NaOH was weighed and dissolved in deionized water. Subsequently, 1.43 mL of acrylic acid (AA) was added dropwise using a pipette while stirring continuously in an ice-water bath. The NaOH solution was used to neutralize the AA under continuous stirring. After neutralization, the mixture was combined with the OSM-Cys solution [49,74], followed by the addition of N,N’-methylenebisacrylamide (MBA) and ammonium persulfate (APS). The resulting mixture was stirred for an additional 15 min to ensure thorough mixing. To remove air bubbles, the solution was placed in a KQ-100 ultrasonic cleaner (water bath, 250 W, 40 kHz) for degassing. Finally, the mixture was polymerized at 353.15 K for 10 min. The entire synthesis process is illustrated in Figure 9. After polymerization, the samples were washed extensively with distilled water to remove any unreacted reagents and by-products. The samples were then dried in a forced-air oven at 70 °C and subsequently ground for further analysis.

OSA/PAA hydrogels were prepared similarly. In brief, AA, NaOH, OSA, cysteine (Cys), MBA, and APS were mixed homogeneously in a beaker. The mixture was then polymerized at 353.15 K for 10 min. Following polymerization, the samples were washed with distilled water and dried in a forced-air drying oven at 70 °C to obtain the final OSA/PAA hydrogels.

### 4.5. Characterization

Fourier transform infrared spectroscopy (FT-IR) was employed to study the presence of chemical functional groups in the hydrogel complexes, with FT-IR spectra measured in the range of 4000–500 cm^−1^. The thermal stability of the hydrogels was analyzed using a thermogravimetric-differential thermal analyzer (TG) (HITACHI STA7300, Tokyo, Japan). The surface morphology of the OSM/PAA composite hydrogel was characterized using scanning electron microscopy (SEM). XPS analysis was performed using monochromatic Al Kα X-ray radiation on Thermo Fisher Scientific ESCALAB250Xi (Waltham, MA, USA). The absorbance of Heavy metal solution was measured using a Shanghai Jinghua Technology Instrument Co., LTD’s 752 UV–VIS spectrophotometer.

### 4.6. Determination of Heavy Metal Adsorption Capacity

An amount of dried hydrogel was added to the prepared 200 mg/L Cu^2+^, Zn^2+^, and Ni^2+^. It was subjected to (600 r/min) magnetic stirring until adsorption equilibrium was reached. The concentration of the remaining Cu^2+^ in solution was determined using a UV absorption spectrophotometer after reaction with DDTC color developer, while XO (Xylenol Orange) was used for the titration of Zn^2+^ and Ni^2+^. The hydrogel adsorption capacity was calculated by the following equation:

(3) $$C_{e}=A\times n\times k$$

(4) $$q_{e}=\frac{\left(V\times C_{o}-V\times C_{e}\right)}{m}$$

where A denotes the absorbance of Cu^2+^, Zn^2+^, and Ni^2+^ in the measured solution, n denotes the dilution of the measured liquid, and k is the slope of the standard curve for Cu^2+^ and other metal solutions; Co (mg·L^−1^) and Ce (mg·L^−1)^ represent the initial and equilibrium concentrations of Cu^2+^, Zn^2+^, Ni^2+^ in solution, respectively. qe (mg·L^−1^) is the amount of Cu^2+^, Zn^2+^, and Ni^2+^ adsorbed on the adsorbent, V (L) is the volume of solution of Cu^2+^, Zn^2+^, and Ni^2+^, while m (g) is the weight of the adsorbent. The average value was taken by repeating three times.

### 4.7. Methods for Determining Adsorption Kinetics and Isotherms

Langmuir, Freundlich, Temkin and Dubinin–Radushkevich isotherm models are the four main models that describe adsorption behavior [75]. The Langmuir model is represented by the following equation:

(5) $$\frac{C_{e}}{q_{e}}=\frac{C_{e}}{q_{m}}+\frac{1}{K_{L}q_{m}}$$

The Freundlich model is represented by the following equation:

(6) $${\mathrm{lnq}}_{e}=\frac{1}{n}{\mathrm{lnC}}_{e}+{\mathrm{lnK}}_{F}$$

The Temkin isotherm model can be expressed as the equation:

(7) $$q_{e}=\frac{\mathrm{RT}}{b_{T}}{\mathrm{lnC}}_{e}+\frac{\mathrm{RT}}{b_{T}}{\mathrm{lnA}}_{T}$$

The DR isotherm model can be expressed as the equation:

(8) $${\mathrm{lnq}}_{e}=-\beta {ℇ}^{2}+{\mathrm{lnq}}_{s}$$

In these equations, the concentration of heavy metal ions at equilibrium is given by C_e_, the number of heavy metal ions adsorbed at equilibrium is denoted qe, and the maximum adsorption capacity of the hydrogel is denoted q_m_. The empirical parameter in the Freundlich model is 1/n, Temkin model b_T_ (J/mol) is the heat of adsorption, q_s_ is the monolayer capacity in DR, and the binding constants for the Langmuir, Freundlich, Temkin and Dubinin–Radushkevich equations are K_L_, K_F_, A_T_, and β, respectively. The Temkin isotherm model is mainly for the case that does not fit the Langmuir isotherm model at high concentrations, while the DR adsorption isotherm model is a model based on the DR equivalent capillary pore model. The model shows the adsorption activity and the distribution of metal ions on the surface through the quantitative description of the DR equation [76].

Quasi-primary and quasi-secondary kinetic models are two commonly used kinetic models for hydrogel adsorption as defined below [77]:

(9) $$q_{t}=q_{e}\left(1-e^{-k_{1}t}\right)$$

(10) $$q_{t}=\frac{q_{e}^{2}k_{2}t}{1+k_{2}q_{e}t}$$

The amount of heavy metal ions adsorbed at equilibrium is denoted qe and the amount adsorbed at any time is denoted q_t_. The quasi-first and quasi-second order rate constants are k_1_ and k_2_.

### 4.8. Recyclability Testing of Hydrogels

In order to evaluate the recyclability of the hydrogels, we performed cyclic adsorption experiments. The adsorbent loaded with metal ions in 2.6 and above was immersed in 25 mL of 0.1 M HCl solution and stirred (600 r/min) for 30 min, and the hydrogel was thoroughly washed with deionized water and reused for the next adsorption process. The above process was repeated 10 times, and the repeatability of this hydrogel was tested by adsorption/desorption cycles.

## Figures and Tables

**Table 1:** Thermodynamic parameters of adsorbent adsorption of Cu^2+^, Zn^2+^, and Ni^2+^ ions.

Thermodynamic Parameter		Temperature (K)		
		298.15	323.15	348.15
Cu^2+^	lnKc	0.3188253	0.320327	0.409905
	ΔG (kJ·mol^−1^)	−0.79031	−0.86061	−1.18648
	ΔS (J·mol^−1^K^−1^)	7.71		
	ΔH (kJ·mol^−1^)	1.54682		
Zn^2+^	lnKc	0.2045785	0.203116	0.350324
	ΔG (kJ·mol^−1^)	−0.507113	−0.54571	−1.01402
	ΔS (J·mol^−1^K^−1^)	9.77		
	ΔH (kJ·mol^−1^)	2.472096		
Ni^2+^	lnKc	0.5941949	0.636596	0.772223
	ΔG (kJ·mol^−1^)	−1.472902	−1.71032	−2.23522
	ΔS (J·mol^−1^K^−1^)	15.03		
	ΔH (kJ·mol^−1^)	3.05538		
$\mathrm{Kc}=\frac{\mathrm{qe}}{\mathrm{Ce}}$	ΔG°= −RTlnKc	$\mathrm{lnKc}=\frac{\left(-{\Delta H}^{o}\right)}{\mathrm{RT}}+\frac{{\Delta S}^{o}}{R}$		

R is the gas constant (8.314 J·mol^−1^K^−1^), T is temperature (K), qe is the equilibrium concentration of adsorbed metal ions (mg/L), and Ce is the equilibrium concentration of metal ions in solution (mg/L).

**Table 2:** Kinetic parameters of adsorbents for adsorption of Cu(II), Zn(II), Ni(II) ions.

	Pseudo First-Order Dynamics			Pseudo-Secondary Dynamics		
	K_1_	q_1_	R_1_^2^	K_2_	q_2_	R_2_^2^
CuSO_4_	171.684	0.0652	0.9877	186.498	0.00061	0.9981
Cu(NO_3_)_2_	89.4957	0.0403	0.9563	106.073	0.00048	0.9949
CuCl_2_	104.495	0.0653	0.9705	146.996	0.00024	0.9815
ZnSO_4_	200.002	0.0168	0.9113	172.662	0.00046	0.9940
NiSO_4_	177.528	0.1065	0.9269	196.153	0.00363	0.9852

**Table 3:** Equations and characteristic parameters of adsorbent adsorption of Cu(II), Zn(II), Ni(II).

Isotherm Model	Isotherm Parameters	Cu(II)	Zn(II)	Ni(II)
Langmuir	q_m_ (mg/g)	367.64	398.4	409.83
	K_L_ (L/mg)	0.0124	0.0088	0.0175
	R^2^	0.81286	0.75149	0.90659
Freundlich	1/n	0.3602	0.4684	0.3829
	K_f_ (L/mg)	36.03	20.08	40.417
	R^2^	0.88065	0.93286	0.96404
Temkin	b_T_ (KJ/mol)	2.404	2.028	1.836
	A_T_ (L/g)	15.95	7.945	2.776
	R^2^	0.74953	0.78991	0.87864
Dubinin–Radushkevich	q_s_ (mg/g)	225.04	228.04	281.25
	β (mol^2^/J^2^)	4.40854 × 10^−8^	7.40329 × 10^−8^	5.12641 × 10^−8^
	R^2^	0.51875	0.65944	0.75081

**Table 4:** Comparison of adsorption capacity of OSM/PAA prepared in this paper with recently reported biosorbents.

Absorbent	Qt (mg/g)			Refer
	Cu(II)	Zn(II)	Ni(II)	
Pal/CLDH	55.8		23.9	[67]
PEI-modified chitosan	128.96		105.98	[68]
Cellulose-chitosan	94.3			[37]
KCTS/PAM	72.39	51.89		[61]
CMC sponge	6.8		7.2	[69]
ECSDNH		160.19	72.67	[70]
PVA-CS/CE		26.74		[71]
OSM/PAA	172.93	162.84	208.52	This work

## Data Availability

Data will be made available on request.

## Supplementary Materials

The following supporting information can be downloaded at https://www.mdpi.com/article/10.3390/gels11040224/s1: Figure S1, Effect of synthesis conditions on the adsorption of OSM/PAA: a. Crosslinker Cys variable relative to SM (%); b. SM fraction; c. Degree of neutralization of AA; d. Initiator content; e. Crosslinker content MBA (all variables except a relative to AA (%)). Figure S2, Adsorption thermodynamic curve of heavy metal ions adsorbed by OSM/PAA. Figure S3, Langmuir, Freundlich, Temkin, and Dubinin–Radushkevich isotherms for OSM/PAA adsorption of heavy metal ions. Figure S4, Effect of mixed metal ions on adsorption. Figure S5, EDS image of SM.
