# Supplementary material for: Preparation and Heavy Metal Adsorption Performance of 2-Aminopyridine-Modified Sodium Alginate/Polyacrylic Acid Hydrogel
Source: Gels. 2025 Mar 21;11(4):224. doi: 10.3390/gels11040224 (PMC12026975; doi:10.3390/gels11040224)
Supplement: Supplementary file 1 [file gels-11-00224-s001.zip › gels-3496988-supplementary.pdf]

## *Supplementary information*

# The adsorption performance of 2-aminopyridine-modified sodium alginate hydrogel on heavy metals

1、 Conditional filter

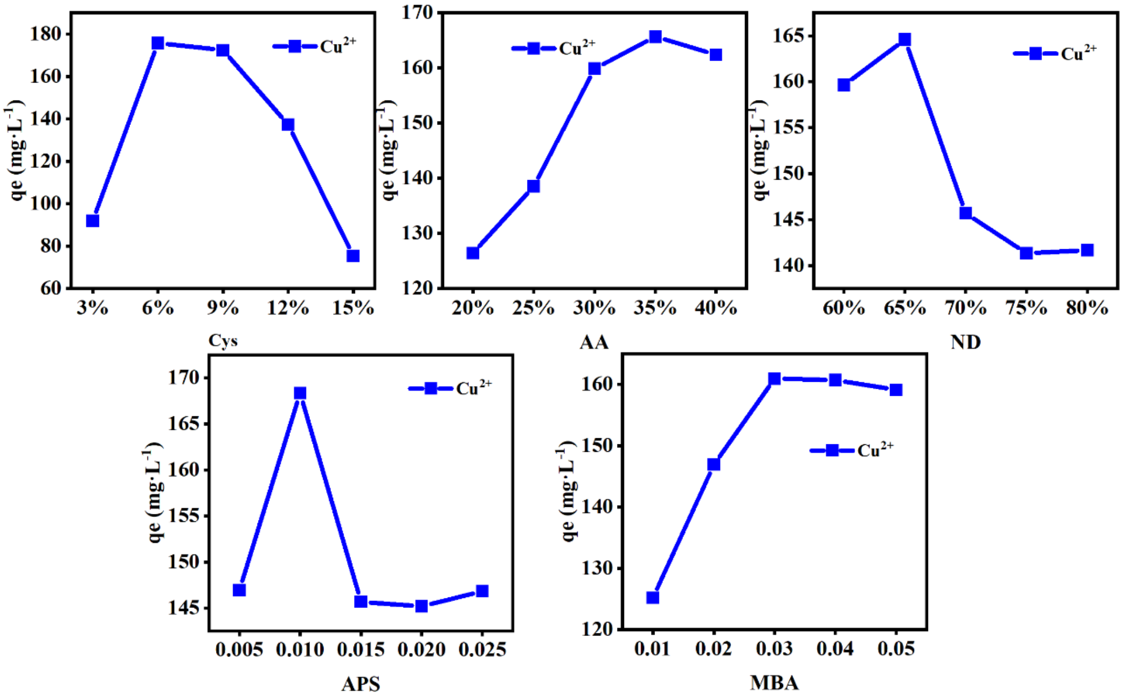


**Figure S1**, Effect of synthesis conditions on the adsorption of OSM/PAA: a. Crosslinker Cys variable relative to SM (%); b. SM fraction; c. Degree of neutralization of AA; d. Initiator content; e. Crosslinker content MBA (all variables except a relative to AA (%)).

2、Thermodynamic curve of heavy metal adsorption by OSM/PAA

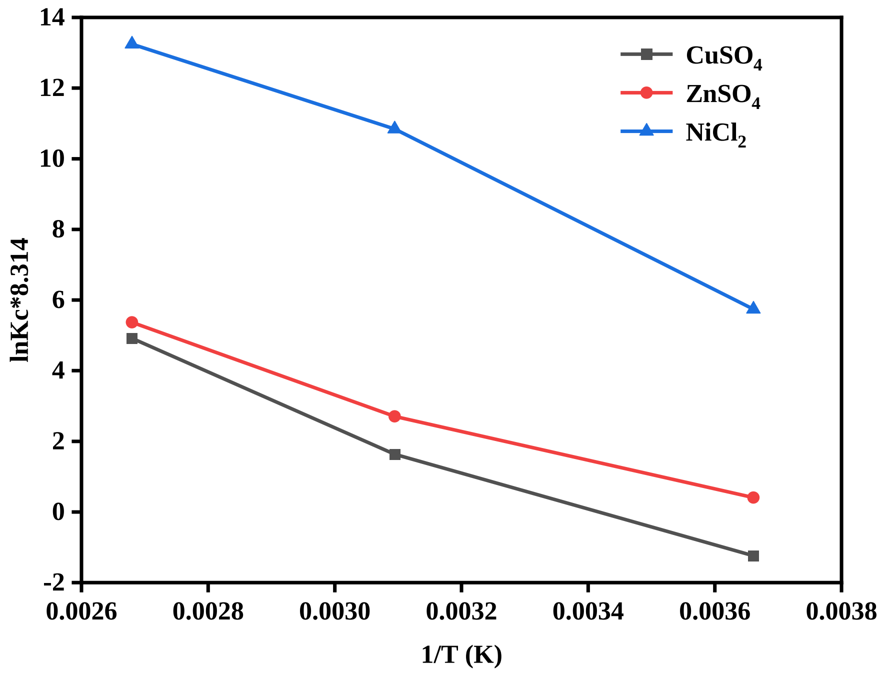


**Figure S2** Adsorption thermodynamic curve of heavy metal ions adsorbed by OSM/PAA

3、Adsorption isotherm

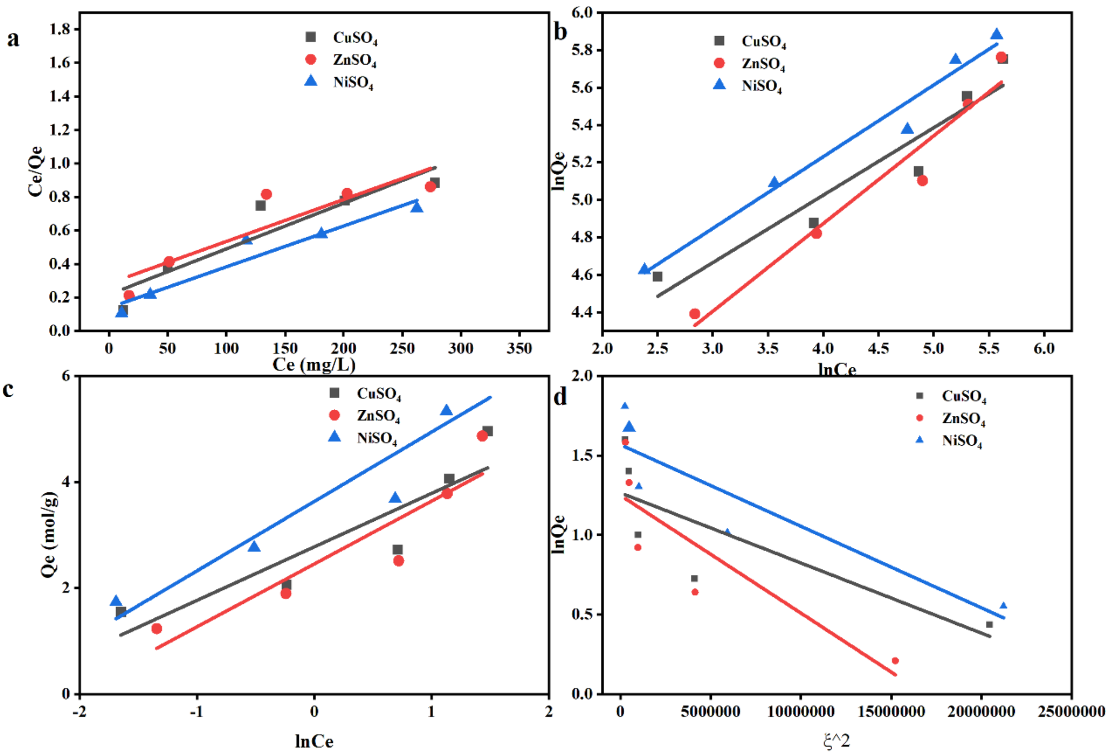


**Figure S3.** Langmuir, Freundlich, Temkin and Dubinin-Radushkevich isotherms for OSM/PAA adsorption of heavy metal ions

4、 Competitive Adsorption

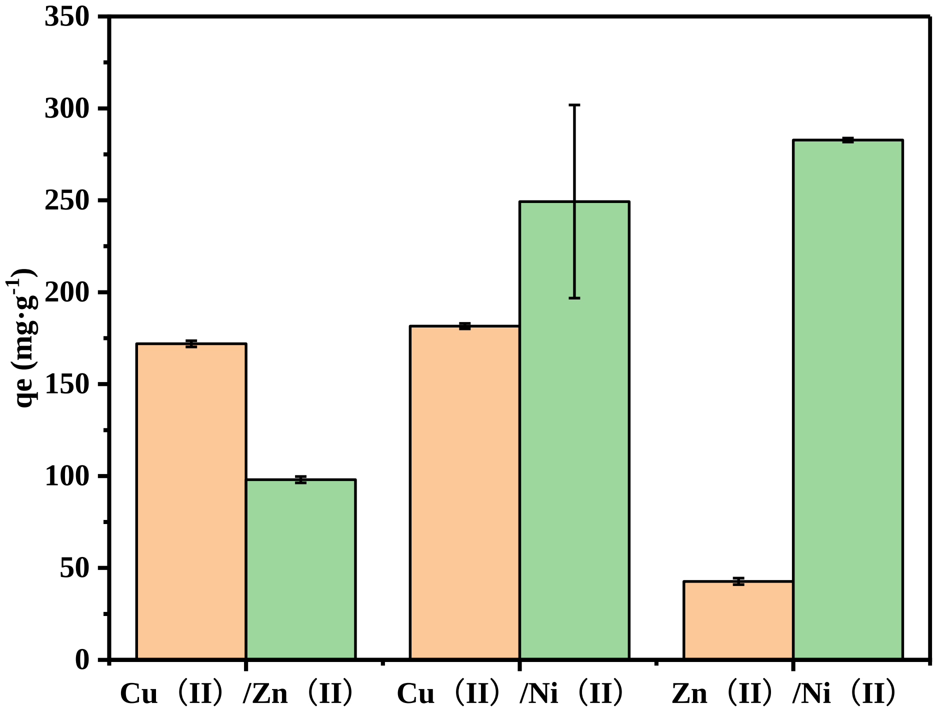


**Figure S4**. Effect of mixed metal ions on adsorption

5、Experimental characterization

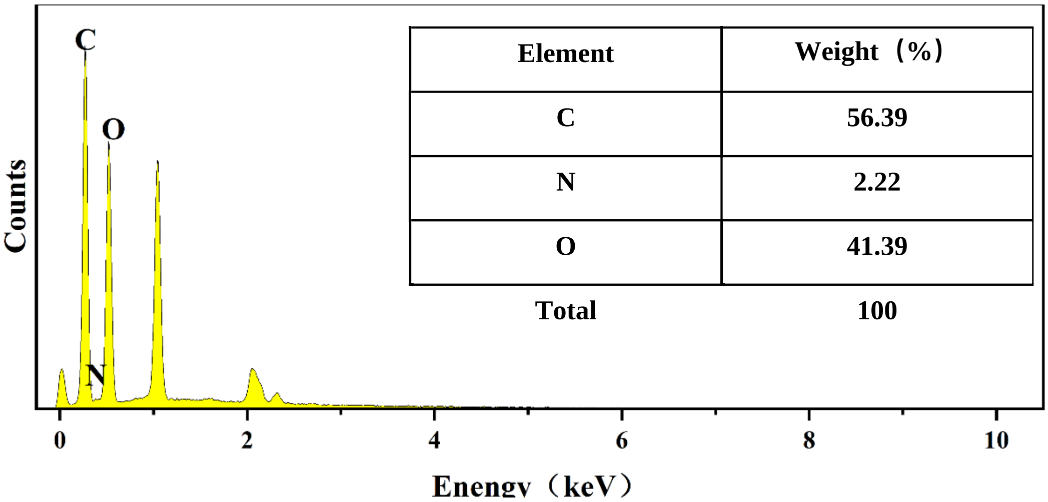


**Figure S5**. EDS image of SM
